# Diffusion of medications for opioid use disorder treatment in jail settings: a convergent mixed methods study of jail staff perspectives

**DOI:** 10.1186/s13722-024-00440-2

**Published:** 2024-02-12

**Authors:** Pryce S. Michener, Elizabeth A. Evans, Warren J. Ferguson, Peter D. Friedmann

**Affiliations:** 1 Clinical and Population Health Research Program, Graduate School of Biomedical Sciences, University of Massachusetts Chan Medical School, 55 Lake Ave North, Worcester, MA 01655 USA; 2 Department of Health Promotion and Policy, School of Public Health and Health Sciences, University of Massachusetts Amherst, 715 North Pleasant Street, Amherst, MA 01003 USA; 3 Department of Family Medicine and Community Health, University of Massachusetts Chan Medical School, 55 Lake Ave North, Worcester, MA 01655 USA; 4 Department of Medicine, University of MA Chan Medical School–Baystate, 759 Chestnut St, Springfield, MA 01199 USA

**Keywords:** Implementation, Jail, Medications for opioid use disorder, Opioid use disorder, Methadone, Buprenorphine, Convergent mixed-methods

## Abstract

**Background:**

Implementation of medications for opioid use disorder (MOUD) in jails varies by facility and across states. Organizational climate, including staff attitudes toward change and exposure to education, can influence perceptions of innovations like MOUD in jails. Using a mixed methods design, we aimed to understand the association between organizational climate and jail staff perceptions of MOUD.

**Methods:**

Jail staff (n = 111) who operate MOUD programs in 6 Massachusetts jails completed surveys that included the Organizational Readiness for Implementing Change (ORIC) survey. Random effects logistic regression models assessed associations between organizational climate and several outcomes of perceived MOUD efficacy, acceptability, and knowledge, while controlling for covariates. Jail staff (N = 61) participated in qualitative interviews and focus groups focused on organizational climate and knowledge diffusion, which we analyzed using inductive and deductive methods.

**Results:**

The results indicate that organizational change readiness on the ORIC was associated with positive perceptions of MOUD, and educational resources facilitated MOUD implementation. Greater ORIC was associated with higher perception of methadone as highly acceptable for jail populations (Odds ratio [OR] 2.3, 95% Confidence Interval [CI] 1.2 to 4.4), and high knowledge of methadone (OR 2.3, 95% CI 1.1 to 4.9), with similar magnitude of effects for buprenorphine. High levels of training for jail staff on methadone and buprenorphine were also associated with higher knowledge of these medications (Methadone: OR 7.2, 95% CI 2.2 to 23.2; Buprenorphine: OR 3.4, 95% CI 1.2 to 9.5). Qualitative results point towards the importance of organizational climate and elucidate educational strategies to improve staff perceptions of MOUD.

**Conclusion:**

Results underscore the importance of organizational climate for successful implementation of jail MOUD programs and provide support for medication-specific educational resources as a facilitator of successful MOUD implementation in jail settings. Findings highlight implementation strategies that may improve jail staff perceptions of MOUD.

**Supplementary Information:**

The online version contains supplementary material available at 10.1186/s13722-024-00440-2.

## Background

Opioid use disorder remains a critical public health issue in the United States, with over 100,000 overdose deaths reported from July 2021 to July 2022 [1]. Projections estimate that over 1.2 million opioid-related deaths will occur between 2020 and 2029 in North America without significant changes in the public health response to opioids [2]. People who have experienced incarceration are especially vulnerable to overdose, with most overdoses occurring within two weeks after release from jail or prison [3, 4]. Overdose is the leading cause of death for people who have recently been released from prison [5]. Medications for opioid use disorder (MOUD), such as methadone and buprenorphine, are the most effective tools to reduce adverse health outcomes related to opioid use and lower the risk of mortality [6]. MOUD care during incarceration has been shown to be associated with decreased post-release mortality, greater engagement with treatment post-release, and reduced illicit substance use [7, 8].

Despite the high risk of mortality for people with opioid use disorder (OUD) who have experienced incarceration, implementation of MOUD in jail settings is limited and highly variable by carceral setting and between regions [9, 10]. Recent studies have documented the inner contextual factors (e.g., organizational and jail leadership treatment philosophy, reentry planning) and outer contextual factors (e.g., state and Medicaid funding, relationship with community treatment providers, social determinants of health) associated with successful implementation of MOUD in jails [11–15]. In Massachusetts, a 2018 legislative mandate established a 4 year pilot program to provide all FDA-approved MOUD in five county jails, with a requirement of incorporating medication maintenance and induction within 30 days prior to release [11]. Two additional county jails joined the pilot program voluntarily. While jails were required to provide MOUD, each facility had wide latitude to tailor MOUD implementation to the specific needs of the jail and its population, including the decision to offer MOUD services in-house or through transport of clients to external MOUD clinics for medication delivery. As a result, the process of implementation of MOUD services in these jails varied highly in terms of staff attitudes and impact on people who were incarcerated at these sites.

Diffusion of innovations theory posits that for an innovation to become ubiquitous within an organization, it must be perceived as both effective and congruent with the organization’s and individuals’ values [16, 17]. Therefore, individuals’ knowledge of an innovation, along with their perceptions of the innovation’s efficacy and acceptability, are key markers of successful diffusion. Diffusion of new interventions may occur through formal processes such as staff training efforts, informal processes such as peer-driven approaches, or a combination of both. Interdisciplinary training for jail staff around MOUD and organizational climate have been identified as key facilitators of MOUD provision in jails [18]. Organizational climate is defined as the collective perception of a work environment by individuals working in that environment, and prior implementation science research has identified organizational climate as impacting workplace attitudes, quality of service delivery, and adoption of new innovations [19–21]. The conceptual model of diffusion of innovations within service organizations further posits that system readiness for change, including organizational climate, is independent of diffusion processes and an important driver of innovation [20]. Organizational readiness for change is a key component of organizational climate, and is defined as the overall capacity for implementing change in an organization [22]. The conceptual model of organizational readiness for change describes two facets of readiness for change: change commitment, defined as organizational members’ collective dedication to change implementation, and change efficacy, defined as organizational members’ collective perceptions of the capacity of the organization to implement change [22, 23].

Recent research has suggested that the perspectives of jail staff about MOUD and the training of jail staff around MOUD administration are critical contextual factors for successful implementation of MOUD in jails [11–15]. Other work has theorized that staff training and education may be able to change attitudes towards an innovation and improve diffusion across an organization [24–26]. However, no universally accepted standard exists for the duration, frequency, or content of training for MOUD delivery in carceral settings. Additionally, some jails choose to educate only those staff who are directly involved in MOUD delivery, while others educate an interdisciplinary cohort of staff including correctional officers and administrators alongside medical and behavioral health staff. Previous work has also found mixed perceptions of the differences between methadone and buprenorphine—on the one hand, some patients and providers believe that methadone is more effective at reducing overdose risk than buprenorphine, but social stigma, racial discrimination, and structural barriers limit its effectiveness [27, 28]. On the other hand, some providers believe that buprenorphine is more effective than or equivalent to methadone, and that the advantages of office-based treatment lead to reduced harm for patients compared to methadone maintenance therapy [29, 30]. Understanding how knowledge and perceptions of different forms of MOUD among jail staff impact their implementation is important for the diffusion and delivery of MOUD in carceral settings.

This study utilizes a mixed-methods convergent design [31] to understand the association between organizational factors and jail staff’s attitudes, beliefs, and knowledge regarding two opioid-agonist medications—methadone and buprenorphine—for people who are incarcerated in jails. We analyze quantitative data from a cross-sectional survey of jail staff across the state of Massachusetts to explore the association between organizational factors, including MOUD-specific training and organizational readiness for change implementation, and staff attitudes towards and knowledge of MOUD treatments. We hypothesize that individuals who received more training on methadone and buprenorphine and work in settings with greater organizational readiness for change will have high markers of successful diffusion of MOUD—perceived efficacy, perceived acceptability, and general knowledge of the evidence base for these medications. We also analyze qualitative data from interviews with jail staff conducted during the early phase of MOUD implementation in these settings to deepen understanding of MOUD perceptions among jail staff.

## Methods

### Parent study

The Massachusetts Justice Community Opioid Innovation Network (MassJCOIN) was established to conduct a Type 1 hybrid effectiveness-implementation study of the pilot program to provide MOUD in seven MA jails [11]. In the present paper, we use a mixed-methods convergent design to analyze quantitative data from staff surveys and qualitative data from focus groups that was collected by MassJCOIN. During the data collection time-period, all participating jails offered buprenorphine on-site and methadone was offered on-site or via transport to nearby community clinics.

### Study design

Jail staff completed a quantitative survey administered from September 2021 to March 2022. This staff survey used established measures of organizational- and individual-level attitudes and philosophies towards implementation of MOUD in criminal justice settings as well as staff knowledge of the evidence base for these medications. MassJCOIN staff conducted focus groups and individual interviews with 61 jail staff employed as clinical, corrections, or senior administrative staff on knowledge production and diffusion of information from December 2019 to January 2020. Details about these qualitative data, including how they were collected, coded, and analyzed, are provided elsewhere [14]. Thus, we describe in detail here only those methods related to the staff survey. All protocols were approved by the Baystate Health Institutional Review Board.

### Study population

The staff survey was administered to all correctional staff in the seven MA jails that were initiating the implementation of a MOUD program. To recruit participants, research staff coordinated with each jail’s MOUD program liaison to identify staff who directly operated the MOUD program or had knowledge of it, including correctional officers, medical and behavioral health clinicians, and administrators. Research staff directly emailed jail staff with information about the study along with a link to complete the survey online. The recruitment email explained that participation would not affect staff employment and that responses would be kept confidential. The survey was emailed to 248 participants. During data collection, one site had a persistently low response due to administrative challenges, and it was decided to drop this site from the staff survey. Thus, of the 209 participants from the remaining six sites who were invited to participate in the survey, 149 participants completed it, for a response rate of 71.29%. Participants were omitted from analysis if they lacked data on key measures (n = 25) (i.e., MOUD efficacy, acceptability, training, knowledge) or covariates (n = 13), leaving an analytic sample of 111 participants. The sociodemographic characteristics of those who were omitted (n = 38) was similar to the analytic sample (n = 111), except that those who were omitted had spent an average of 3 years longer in their current position compared to those in the analytic sample.

### Independent variables

MOUD-specific training is measured separately for methadone and buprenorphine on a 7-point ordinal scale, where 1 indicates low levels of training and 7 indicates high levels of training for each MOUD type [26]. The survey asked respondents: “To what extent have you received specific training about the following?” with separate questions for methadone and buprenorphine. Training was dichotomized as “High training” if participants responded with a score ≥ 6 for these measures to capture participants with high levels of training and education around MOUD compared to those with less training.

System readiness for change has been described in implementation science literature as a key contributor and driver of the adoption of new innovations [20]. Organizational philosophy and readiness for change were calculated from the Organizational Readiness for Implementing Change survey (ORIC) composite score and component scores [22, 23]. The ORIC is composed of two measures, Change Commitment and Change Efficacy, which reflect different aspects of organizational readiness for change. Change Commitment refers to whether members of the organization consider an intervention to be beneficial, worthwhile, and/or necessary, while Change Efficacy refers to organizational members’ perceptions of an intervention’s feasibility given available resources [22]. The ORIC includes 10 questions measured on a 5-point ordinal score with 5 indicating high organizational readiness for change. For instance, the survey asked respondents to rate their agreement with the statement: “People who work here are motivated to implement the Jail MAT program” as one component of Change Commitment and “People who work here feel confident that they can handle the challenges that might arise in implementing the Jail MAT program” as a measure of Change Efficacy. Other covariates included in the model were staff position (behavioral health, medical/nursing, correctional, and administrative) and time spent in position.

### Dependent variables

Two key outcomes were *perceived efficacy* and *perceived acceptability*, measured separately, for methadone and buprenorphine on a 1–7 ordinal scale where 7 indicates a highly favorable view [26]. As an example, respondents were asked “In your opinion, how acceptable is each of the following medications for the treatment of opioid use disorder with justice involved populations?” Responses were dichotomized as “High perceived efficacy” and “High perceived acceptability” if participants responded with a score ≥ 6 for these measures. These cut-off values were chosen to reflect participants who perceived MOUD to be highly efficacious and acceptable, which is indicative of successful diffusion of the MOUD intervention.

A third key outcome was *knowledge diffusion*, measured separately for methadone and buprenorphine, via 19 items assessing correctional staff’s understanding of the evidence base for treatment on a 0–5 ordinal scale, where 5 indicates higher familiarity [32]. Questions centered around MOUD effectiveness at reducing opioid users’ risk of death and other adverse health outcomes, among other topics [32]. Scores were dichotomized as “High knowledge” if participants had an average score of ≥ 4. This cut-off value was chosen to capture participants with a high degree of knowledge of MOUD, which is indicative of successful diffusion of knowledge.

### Quantitative analysis

Descriptive statistics were utilized to characterize survey respondents. We used mixed effects logistic regression models to assess associations between organizational factors and each outcome—high perceived efficacy, high perceived acceptability, and high knowledge—while controlling for covariates and accounting for random effects of jail site. Regression analyses were guided by directed acyclic graph (DAG) construction to determine the minimum sufficient adjustment set of confounders necessary to estimate the effect of independent variables on the primary outcomes [33]. The DAG was constructed prior to analysis and was revised through discussion with the team of MassJCOIN investigators (Additional file 1). The DAG was informed by the conceptual models of diffusion of innovations and organizational readiness for change. It is important to note that the DAG is informed by prior knowledge, represents investigators’ hypotheses about the causal relationship between variables, and is used as a tool to guide identification of potential confounders and inform analyses [34]. Regression models were tested using each component measure for organizational philosophy/capacity for change instead of the overall summary score, and outcomes between models using these different component variables were compared to assess robustness of the analysis at a threshold of significance (p < 0.05). Sensitivity analyses were performed without dichotomization of dependent and independent variables to ensure that dichotomization did not bias results. All analyses were completed using Stata 15.1 [35].

### Qualitative analysis

Methods describing the interview guide and qualitative analysis protocol have been published previously [14]. Research staff conducted interviews using the Exploration, Preparation, Implementation, and Sustainment (EPIS) theoretical framework to construct the interview guide and frame analyses, and inductive and deductive coding methods were applied [18, 36]. The EPIS framework categorizes organizational factors into Inner and Outer Contexts as well as bridging factors which link Inner and Outer Context constructs [18]. Codes that were gathered under the Inner Context EPIS construct of “Staff Perspectives and Hiring Processes” and “Staff Training” were analyzed to capture staff perceptions of organizational climate and training/education around MOUD. These qualitative data have been examined in prior qualitative research, although the codes used for this study have not been previously analyzed [12–15]. Qualitative themes were identified deductively. The site which was excluded from quantitative survey analysis was also excluded from qualitative analysis.

## Results

Survey respondents had a mean age of 44, a majority were women, and the great majority were white participants (Table 1). Most (77%) staff had attained at least a bachelor’s degree or higher educational degree. Staff worked for an average time of 5.4 years in their respective roles. Proportions of staff surveyed were representative of the population of jail staff who are stakeholders in MOUD implementation, with correctional officers making up the highest proportion of participants at 36%.

**Table 1:** Demographic data of survey participants

Variable	Total (N = 111)
Age	44.28 (11.12)
N (% Missing)	109 (1.8%)
Gender	
Male	46 (41.4%)
Female	61 (55.0%)
Prefer Not to Answer	4 (3.6%)
Race	
American Indian/Alaska Native	1 (0.9%)
Black or African American	4 (3.6%)
White	92 (82.9%)
More than one race	2 (1.8%)
Other	7 (6.3%)
Prefer Not to Answer	4 (3.6%)
Did Not Respond	1 (0.9%)
Hispanic or Latinx Ethnicity	
No	100 (90.1%)
Yes	10 (9.0%)
Did Not Respond	1 (0.9%)
Staff job category	
Behavioral Health Treatment	36 (32.4%)
Medical/Nursing	29 (26.1%)
Correctional	40 (36.0%)
Administrative	6 (5.4%)
Staff time in role (years)	5.40 (5.43)
Education	
High school diploma or equivalent	3 (2.7%)
Some college, but no degree	10 (9.0%)
Associate’s degree	12 (10.8%)
Bachelor’s degree	44 (39.6%)
Master’s degree	37 (33.3%)
Doctoral degree or equivalent	4 (3.6%)
Other	1 (0.9%)
Did Not Respond	3 (2.4%)

Continuous variables are presented as mean (standard deviation) and categorical variables are presented as N (percent of total)

### Perceptions of MOUD training, efficacy, acceptability, and knowledge

Approximately 43% of participants reported having received a high-level of training for methadone and 47% had received a high-level of training for buprenorphine (Table 2). Regarding outcome indicators, methadone was reported to be highly efficacious by 43% of participants, while buprenorphine was reported as highly efficacious by 32% of participants. Methadone was rated by 39% of participants as being highly acceptable for treatment of incarcerated populations, whereas buprenorphine was reported as highly acceptable by 32% of participants. Similar proportions of participants had “high knowledge” of methadone (29%) and buprenorphine (30%).

**Table 2:** Independent and dependent variables

Variable	Total (N = 111)
To what extent have you received specific training about the following: (Scale: 1–7)	
Methadone	4.99 (1.70)
Buprenorphine	5.16 (1.60)
High methadone-specific training (Training score ≥ 6)	
Low training	63 (56.8%)
High training	48 (43.2%)
High buprenorphine-specific training (Training score ≥ 6)	
Low training	59 (53.2%)
High training	52 (46.8%)
Organizational Readiness for Implementing Change (ORIC) (Scale: 1–5)	
Overall	4.11 (0.80)
Change Commitment	4.22 (0.81)
Change Efficacy	4.00 (0.90)
Based on your knowledge and personal experience, to what extent do you consider each of the following medications for opioid use disorder to be effective with justice involved populations: (Scale: 1–7)	
Methadone	4.76 (1.73)
Buprenorphine	4.63 (1.58)
High methadone efficacy score (Efficacy score ≥ 6)	
Low efficacy	63 (56.8%)
High efficacy	48 (43.2%)
High buprenorphine efficacy score (Efficacy score ≥ 6)	
Low efficacy	76 (68.5%)
High efficacy	35 (31.5%)
In your opinion, how acceptable is each of the following medications for the treatment of opioid use disorder with justice involved populations: (Scale: 1–7)	
Methadone	4.64 (1.88)
Buprenorphine	4.63 (1.69)
High methadone acceptability score (Acceptability score ≥ 6)	
Low acceptability	68 (61.3%)
High acceptability	43 (38.7%)
High buprenorphine acceptability score (Acceptability score ≥ 6)	
Low acceptability	76 (68.5%)
High acceptability	35 (31.5%)
Knowledge of MOUD evidence base: (Scale: 1–5)	
Methadone	3.53 (0.74)
Buprenorphine	3.56 (0.74)
High methadone knowledge score (Knowledge ≥ 4)	
Low knowledge	79 (71.2%)
High knowledge	32 (28.8%)
High buprenorphine knowledge score (Knowledge ≥ 4)	
Low knowledge	78 (70.3%)
High knowledge	33 (29.7%)

Continuous variables are presented as mean (standard deviation) and categorical variables are presented as N (percent of total)

### Associations between organizational factors and efficacy, acceptability, and knowledge

#### Organizational readiness for change

The total ORIC score was significant across all models for both methadone and buprenorphine and was associated with high perceived efficacy, acceptability, and knowledge. Increases in organizational readiness for change were associated with 2- to threefold higher odds that participants would report methadone (Table 3) and buprenorphine (Table 4) as highly acceptable and highly efficacious for incarcerated populations and an equivalent increase in the odds that participants would have high knowledge of each medication.

**Table 3:** Outcome of mixed effect methadone regression models

Methadone	High efficacy	High acceptability	High knowledge
	Odds ratio (95% Confidence Interval)	Odds ratio (95% Confidence Interval)	Odds ratio (95% Confidence Interval)
*Organizational Readiness for Change:*			
ORIC–Total	**2.15 (1.08 to 4.27)**	**2.26 (1.15 to 4.44)**	**2.28 (1.06 to 4.89)**
ORIC–Change Commitment	1.63 (0.85 to 3.11)	**1.98 (1.03 to 3.83)**	1.76 (0.85 to 3.61)
ORIC–Change Efficacy	**2.36 (1.23 to 4.54)**	**2.20 (1.17 to 4.12)**	**2.56 (1.21 to 5.40)**
*Training:*			
Methadone Training (High)	**4.73 (1.63 to 13.73)**	**3.21 (1.15 to 8.91)**	**7.18 (2.22 to 23.21)**
*Other variables:*			
Staff Role			
Behavioral Health	*Reference*	*Reference*	*Reference*
Medical/Nursing	2.33 (0.66 to 8.25)	2.27 (0.67 to 7.64)	2.08 (0.57 to 7.59)
Correctional	**4.06 (1.02 to 16.20)**	2.41 (0.67 to 8.69)	0.79 (0.19 to 3.21)
Administrative	3.55 (0.36 to 35.17)	**20.99 (1.67 to 264.33)**	1.83 (0.18 to 18.31)
Staff Time in Role	1.04 (0.94 to 1.15)	0.96 (0.87 to 1.06)	0.93 (0.82 to 1.04)

Odds ratios reported from ORIC—Total model, though odds ratios are similar for ORIC Change Commitment and Change Efficacy models. Bold text indicates significance at p > 0.05

**Table 4:** Outcome of mixed effect buprenorphine regression models

Buprenorphine	High efficacy	High acceptability	High knowledge
	Odds ratio (95% Confidence Interval)	Odds ratio (95% Confidence Interval)	Odds ratio (95% Confidence Interval)
*Organizational Readiness for Change*			
ORIC–Total	**2.43 (1.20 to 4.90)**	**2.73 (1.17 to 6.38)**	**2.63 (1.25 to 5.52)**
ORIC–Change Commitment	**2.20 (1.11 to 4.36)**	**2.54 (1.11 to 5.81)**	1.92 (0.97 to 3.81)
ORIC–Change Efficacy	**2.26 (1.16 to 4.40)**	**2.42 (1.11 to 5.29)**	**2.90 (1.42 to 5.92)**
*Training:*			
Buprenorphine FTraining (High)	2.05 (0.81 to 5.22)	**5.23 (1.56 to 17.52)**	**3.40 (1.21 to 9.52)**
*Other variables:*			
Staff Role			
Behavioral Health	*Reference*	*Reference*	*Reference*
Medical/Nursing	0.64 (0.20 to 2.08)	1.60 (0.41 to 6.29)	2.02 (0.60 to 6.78)
Correctional	0.84 (0.26 to 2.69)	2.65 (0.59 to 11.94)	0.30 (0.08 to 1.11)
Administrative	0.89 (0.12 to 6.44)	**57.29 (3.15 to 1041.54)**	1.08 (0.14 to 8.46)
Staff Time in Role	0.96 (0.87 to 1.05)	0.90 (0.80 to 1.01)	0.89 (0.80 to 1.00)

Odds ratios reported from ORIC—Total model, though odds ratios are similar for ORIC Change Commitment and Change Efficacy models

Bold text indicates significance at p > 0.05

When broken down into component scores of Change Commitment and Change Efficacy, the association between training and perceptions of MOUD held. Change Commitment was associated with nearly twofold higher odds that participants would report methadone as highly acceptable (95% CI1.03 to 3.83), but Change Commitment was not significantly associated with either efficacy or knowledge about methadone. For buprenorphine, Change Commitment was associated with both high perceived efficacy and high perceived acceptability at odds ratios of 2.20 (95% CI 1.11 to 4.36) and 2.54 (95% CI 1.11 to 5.81), respectively. Change Efficacy was significant across all tested models, and increases in Change Efficacy were associated with 2- to threefold higher odds of participants reporting high efficacy, acceptability, and knowledge of both methadone and buprenorphine. The importance of change commitment and efficacy were supported by qualitative findings which highlighted these domains as important drivers of change.

Staff commitment to change was identified by participants as a key facilitator of implementation of new MOUD programs in jails. When asked what factors led to success in jail MOUD treatment programs, one participant responded, “…you have to have people that are committed to it and believe in it…I think that's really important to empowering the people that you're involving. And you have to involve people that are doing the work…The decisions and the input has to be made with the folks that are on the ground level…doing the work” (ID-6, P2). Change efficacy was also identified as an important driver of organizations’ ability to successfully implement MOUD programs in jails. A common theme that emerged from staff interviews was the need for support from grants and the state legislature to financially support the MOUD program in order to continue providing treatment services: “So it’s the cost of clinicians, the cost of medical staff, the cost of all that…those aspects that [are] being paid for now under grants, that if it’s going to continue, we’re going to have to absorb that, so there’s going to have to be some give and take with the legislature to say…you’re going to need so much more…in your budget to continue this” (ID-23, P2). Another participant said, “we have to have…a way to pay for it, and the only way to pay for it is for the legislature…to put it into some sort of a codified system that…we can work with” (ID-20). Both change commitment and change efficacy were identified as synergistic factors facilitating successful implementation of jail MOUD programs. As one participant in a focus group observed that “having the legislature in place certainly helped” implementation of the MOUD program, another focus group participant agreed, saying “having that backing now in addition to…already knowing that we wanted to pursue this was really helpful in changing people’s opinions” (ID-22, P, P4). Taken together, these findings highlight how change efficacy and change commitment are mutually supportive and intersect to produce an environment conducive to successful implementation of jail MOUD programs.

#### Perceived efficacy

A high level of methadone training was associated with nearly fivefold higher odds of reporting high perceived efficacy of methadone (OR: 4.7, 95% CI 1.63 to 13.73) after adjusting for other factors (Table 3). A similar but smaller effect was evident for buprenorphine, but this association was not statistically significant (OR: 2.05, 95% CI 0.81 to 5.22) (Table 4).

The importance of ongoing training and education for jail staff around MOUD has been previously reported as a key facilitator of successful MOUD implementation [14]. The qualitative results presented here support that staff training and education improves perceived efficacy of methadone and buprenorphine. One focus group participant described the importance of having open conversations with jail staff as an educational tool to improve perceived efficacy of MOUD:

“It’s encouraging an open dialogue…especially…men and women [officers] that are in the housing units and…on that frontline…the staff that's…in this everyday asking questions about, ‘why are we doing this? How is this helpful? Why is this evidence-based…effective treatment’ when they may not even know what that means?…Just getting staff educated on what those meant…And, why is this important and why is this…the direction that our department is going, and really, our communities are going in? I think that was the big piece. Encouraging open dialogue…some officers asked some really great questions just because, again, they didn't know, and to ask questions and kind of share opinions about it was helpful” (ID-6, P10).

Another participant reported “I think…there’s been…overall spread of education on [MOUD]…from community providers coming in to us and…educating a lot of our staff on why it’s the standard of care… ‘cause I think especially our security staff has only really seen people not do well on it. Those are…their personal experiences. People haven’t done well on it. They keep coming and coming back. So why would we offer it to them when we know they’ve reported they’ve abused it….So I think that spread of education, we’ve seen a major shift since that” (ID-26).

Taken together, these qualitative results support the quantitative results, and highlight how and why education and training of jail staff impacted perceived efficacy. Specifically, training was described as an ongoing process, rather than a single event, that helped to identify gaps in staff knowledge, provide a space for genuine dialogue, and achieve staff understanding that MOUD in correctional settings was supported by jail leadership and also by the broader community. Training was reported to change staff perceptions toward greater understanding of MOUD as an evidence-based and effective treatment for carceral populations.

#### Perceived acceptability

High levels of staff training were associated with greater perception of methadone and buprenorphine as acceptable for treatment of incarcerated populations. Participants with high methadone-specific training were 3.2 times more likely to report high methadone acceptability (95% CI 1.15 to 8.91), while participants with high buprenorphine-specific training were 5.2 times more likely to report high buprenorphine acceptability (95% CI 1.56 to 17.52).

Survey findings were supported by qualitative data which indicated that training was related to improved staff perceptions of MOUD acceptability for incarcerated populations. One participant reported that training helped staff understand the process of MOUD treatment and overcome the belief that MOUD treatment was substituting one addiction for another:

“Sometimes I…heard people saying…they felt that we were offering this medication as a substitute for another…like it could turn into another addiction…that’s something I consistently heard when people didn’t get much education surrounding it. Until…they realized…we’re not just dependent on giving the client a medication and just sending them on their way. There’s other components to it. There’s the counseling component and working on…changing…thinking patterns and then to supplement it… with a medication… I think…once people got that explanation, it was a little bit more palatable. They understood that it’s more of a package. It’s not just, here take this medication, you’re gonna be cured” (ID-22).

Although many staff were skeptical based on personal experiences with OUD, this participant reported that education helped overcome that barrier. Another participant in a focus group said that education motivated staff to better understand the value systems that had led to the implementation of MOUD:

“I taught a couple sessions of our [training] on MAT program. Going from somebody who was skeptical…and then at the very end, they're like, "Thank you. You educated me so much more on the topic." They felt like they had that definite buy in…they were like, "Okay, this makes sense. Now that you've explained it to me, this really makes sense. All right, this is good that we're going in this direction as a group, as a team." I saw that. I observed that. I had people coming up to me afterwards, after I presented, saying, "Yeah…that really makes sense. I'm glad the sheriff, they're on board with this, because it's going to help so many people out." It was good. It was a good thing” (ID-6, P9).

These qualitative data support that training and education for MOUD improves staff perceptions of these medications as acceptable within the jail setting and consistent with the broader organizations’ values. Specifically, these quotes highlight how education helped to overcome common misconceptions about MOUD treatment, such as the belief that MOUD substitute one addiction for another, which led to a greater perception of these medications as acceptable for incarcerated populations and led to increased buy-in from jail staff. These data support evidence from the quantitative survey which indicated that a high degree of training was associated with high perceived acceptability of MOUD in jail settings.

#### Knowledge

High levels of staff training were associated with high knowledge of both methadone and buprenorphine. High levels of methadone-specific training were associated with a sevenfold higher odds of scoring high knowledge for methadone (95% CI 2.22 to 23.21). High buprenorphine-specific training was associated with 3.4 times greater odds of scoring high knowledge of buprenorphine (95% CI 1.21 to 9.52).

Qualitative findings suggested that knowledge diffusion around MOUD is important for successful implementation. One participant described how a community partner helped convince correctional officers of the importance of MOUD, saying, “I think the education part of it really got rid of the pushback” (ID-5). While education and knowledge production in formal training settings was described as important, informal knowledge transmission, such as conversations with peers and observation of community and correctional partners, were critical to the spread of knowledge around MOUD. One participant described their process for educating staff using informal conversations: “We went over all the myths [of MOUD treatment] as well and debunked the myths and dissected each one and had a conversation around each one, as well… We really dissected all of that, and folks were able to talk about it… It just really helped support the environment and it helped kind of prevent that kind of judging and hierarchy” (ID-6, P5). Another participant described how informal conversations allowed for re-framing concepts around MOUD delivery and improving understanding of these medications: “it opened up some staffs’ eyes. Some staff actually reported, when I did a training last spring and I really broke that down… it was very elucidating for them…being really explicit about the language… Not just re-framing…really breaking it down, what it means” (ID-22).

Education, whether through formal trainings or informal conversations, provided jail staff with an opportunity to re-frame pre-existing beliefs and misconceptions around MOUD and improve knowledge of these medications. These data highlight the importance of bidirectional conversations between jail staff and those who are educating, which can allow for dialogic knowledge production and dissemination that promotes increased staff knowledge of MOUD. These qualitative findings support that diffusion of knowledge should not be strictly limited to formal trainings and that knowledge sharing during informal settings may be vital to improving implementation of MOUD in jail settings.

## Discussion

This study found that greater organizational readiness for change and high levels of MOUD-specific training were associated with jail staff members’ perceptions of methadone and buprenorphine as effective and acceptable medications for people who are incarcerated in jails, indicative of successful diffusion of MOUD in this setting. High levels of training and greater organizational readiness for change were further correlated with greater knowledge of these medications, indicating that organizational climate is associated with better understanding of the rationale and evidence for MOUD treatment among jail staff. These results support the conceptual model of diffusion of innovations in health services organizations that describe organizational readiness of change and diffusion efforts as synergistic and contributory factors towards successful adoption of an innovation [20]. This mixed-methods study supports prior research that has theorized that training and education for organizational staff is critical to improving diffusion of innovations within that organization [24–26].

Qualitative findings align with the quantitative results and indicate that education for jail staff around MOUD was also associated with greater acceptance of and positive perceptions of medication treatment among jail staff. Our prior work indicated that jail staff were largely supportive of training around MOUD and believed that sustained education was crucial to the success of the program [14]. Other research has further shown that education may reduce stigma around MOUD and improve perceptions of these medications [37]. Taken together, the findings from this study build on this previous research to understand the impact of knowledge diffusion in jail settings and provide quantitative evidence that training and education do promote positive perceptions of MOUD among jail staff. This study provides additional support for education that is tailored to specific MOUD types and reactive to the needs of each individual and jail setting.

While education and trainings are important for dissemination of knowledge among jail staff, our findings indicate that organizational readiness for change was a key component of individual perceptions of MOUD. Organizational capacity for change as measured by the ORIC summary score was strongly associated with higher diffusion of knowledge across all tested models. These findings support previous research which found that organizational climate and openness to change were important for the adoption of new innovations [38, 39]. Organizational readiness for change theory describes how implementation of changes is dependent on organizational commitment to change (i.e., do organizational members value the innovation) and change efficacy (i.e., do organizational members consider the innovation to be feasible given available resources) [22]. Our findings suggest that although both components of organizational readiness for change are important, Change Efficacy was associated with higher MOUD knowledge and higher perceptions of both methadone and buprenorphine as highly acceptable and efficacious, indicating that Change Efficacy may be critical for the diffusion of MOUD interventions within carceral settings. These results support the finding that for MOUD interventions to be implemented effectively, organizations must provide financial, material, technological and human resources to support organizational members in implementing changes and to build the change efficacy of the organization.

Organizational readiness for change is likely driven by multiple contextual and organizational factors, including training and education for staff. Although formal trainings and official education are important, our findings indicate that peer-to-peer education through casual conversations, organizational team meetings, or 1-on-1 discussion was another important mode of education for jail staff around MOUD. This finding supports prior qualitative work that suggested that peer learning is critical for the development and sustainment of jail MOUD programs [40]. While direct peer-to-peer learning was not assessed in the quantitative survey, peer support for an innovation is an important component of Change Commitment according to the ORIC [22]. Peer learning may therefore be intrinsically linked with organizational readiness for change. Peer knowledge sharing may be supplementary and complementary to more formal educational modalities and may be a key facilitator for jail MOUD program success. Whether education is driven by peer learning or formal educators, the best opportunities for learning were described as dialogic and nonjudgmental, providing staff with a space to discuss misconceptions and myths around MOUD and arrive at a greater understanding of these medications through shared experiences and conversations. Additionally, a survey of US jails revealed that there is high demand among jails for MOUD education that targets not just correctional officers and other jail staff, but community stakeholders including politicians, judges, clinicians, and other key stakeholders [41]. Results from our survey support that education should be an ongoing process that targets all relevant stakeholders.

This study has several limitations. Its cross-sectional design study prevents us from determining causality and directionality of the relationship between organizational readiness for change and staff perceptions of MOUD. Although it seems likely that greater training results in more knowledge and functional perceptions of MOUD, it might be the converse–that positive attitudes and greater knowledge leads one to participate in training—or the relationship may be bidirectional. As degree of training was measured subjectively in this study, future work should seek to understand which modalities of training are most effective at improving the adoption and diffusion of MOUD in carceral settings. The study also occurred in jails that had already signed on to a legislatively mandated pilot implementation of all three FDA-approved forms of MOUD in Massachusetts; thus, the staff in these jails might not be representative of jail staff nationwide. Quantitative survey data were collected approximately two years after qualitative data were collected, meaning there is the potential that perceptions of MOUD changed over time. In order to minimize this effect, broad organizational attitudes were captured rather than individual attitudes, which may be more susceptible to temporal effects. Future work will assess how qualitative perceptions among jail staff involved with MOUD delivery may have changed temporally during different phases of MOUD implementation. Finally, some participants were excluded from analyses due to missing data. These participants’ demographics were similar to participants who were included, except that those who were excluded worked in their current position for an average of 3 years longer. It is possible that increased time spent working in jail settings contributed to survey fatigue, making it less likely that staff would respond to our survey compared to staff who started in their current position more recently.

## Conclusions

Diffusion of innovations theory has been used to understand the implementation a variety of innovations around MOUD, such as substance use counselors’ attitudes towards buprenorphine and facility- and state-level factors that contribute to successful MOUD implementation [26, 42]. According to diffusion of innovations theory, for an intervention to become widespread in an organization it must first be perceived as both efficacious and acceptable within the organizations’ value system [16, 17]. Our study supports that education and training of an organizations’ members are critical to improving these perceptions and facilitating uptake of an intervention. Organizational readiness for change is also crucial for the diffusion of interventions with an organization, and our study highlights how organizational philosophy and capacity for change can influence individual members of that organization to be more accepting of interventions. Prior implementation studies of MOUD in jail settings have highlighted the importance of training for jail staff and education as a key facilitator of successful implementation of MOUD in this setting [14, 18]. Our study adds to this prior research by providing support for medication-specific educational resources as a facilitator of successful MOUD implementation in jail settings. Results from this mixed-methods analysis of survey and qualitative data of jail staff support this prior research and underscore the importance of organizational climate for successful implementation of jail MOUD programs.

### Supplementary Information

**Additional file 1. **Directed acyclic graph (DAG). Description: DAG model of mixed effect regression model indicating minimum sufficient adjustment set to estimate the effect of MOUD-specific training on MOUD diffusion and staff attitudes towards MOUD. Produced using DAGitty software [32].

## Data Availability

The datasets analyzed during the current study are components of the larger MassJCOIN study and are not currently publicly available. Upon completion of the MassJCOIN study, de-identified data and codebooks will be warehoused and available through the JCOIN Data Commons website (https://jcoin.datacommons.io) [43]. This data sharing plan will comply with the requirements of the privacy rule and federal regulations under HIPAA and 42 CFR Part 2 that govern the protection of individually identifiable information. Data are currently available from the corresponding author on reasonable request.

## Abbreviations

MOUD: Medications for opioid use disorder
OUD: Opioid use disorder
MA: Massachusetts
MassJCOIN: Massachusetts Justice Community Opioid Innovation Network
ORIC: Organizational Readiness for Implementing Change
DAG: Directed acyclic graph
EPIS: Exploration, Preparation, Implementation, and Sustainment
