# Supplementary figures and images for: Diffusion of medications for opioid use disorder treatment in jail settings: a convergent mixed methods study of jail staff perspectives
Source: Addict Sci Clin Pract. 2024 Feb 12;19:10. doi: 10.1186/s13722-024-00440-2 (PMC10863078; doi:10.1186/s13722-024-00440-2)

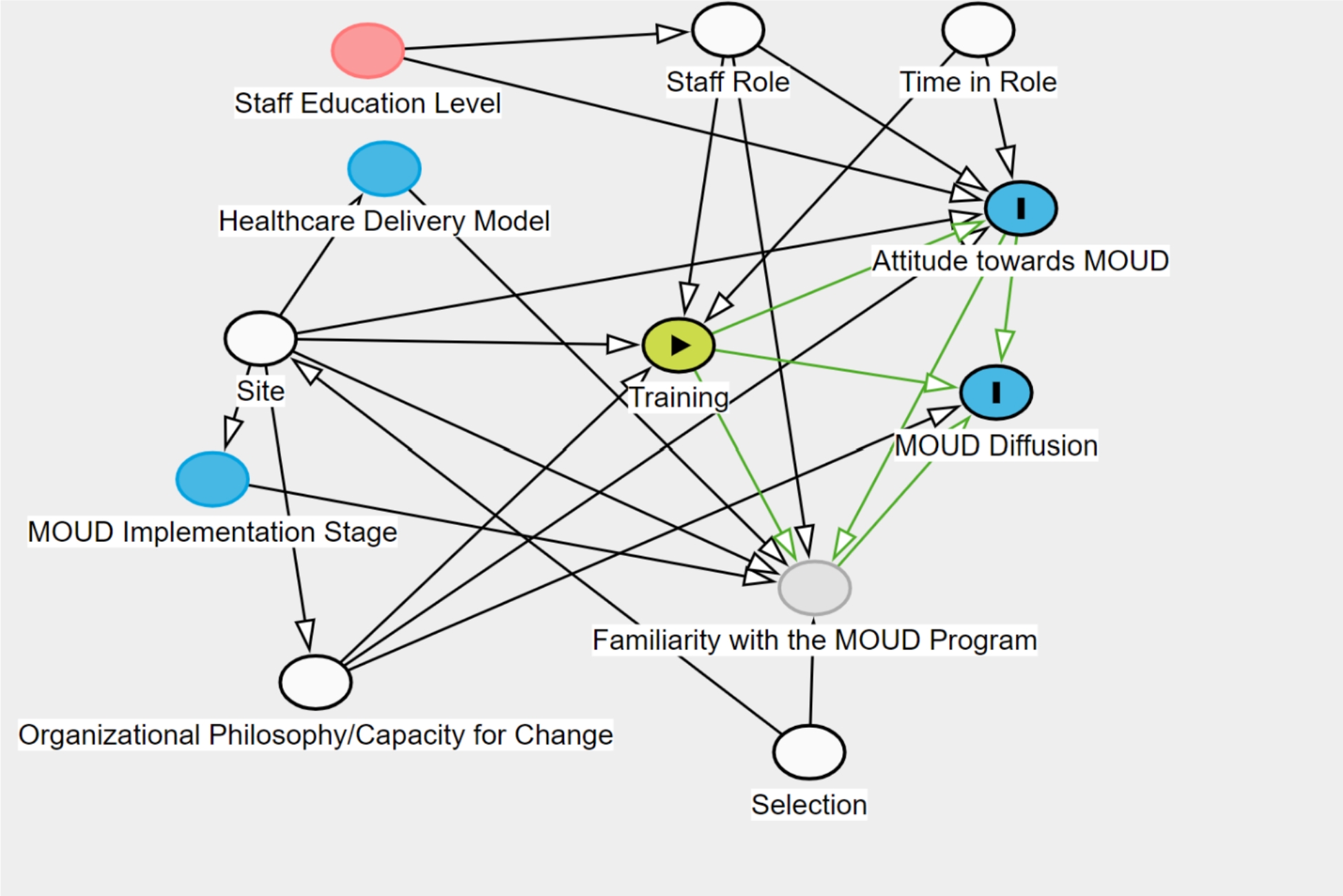

Supplement: Supplementary file 1 — Additional file 1. Directed acyclic graph (DAG). Description: DAG model of mixed effect regression model indicating minimum sufficient adjustment set to estimate the effect of MOUD-specific training on MOUD diffusion and staff attitudes towards MOUD. Produced using DAGitty software [32]. [file 13722_2024_440_MOESM1_ESM.jpeg]
